# Validity of SF-12 summary scores in a Greek general population

**DOI:** 10.1186/1477-7525-5-55

**Published:** 2007-09-28

**Authors:** Nick Kontodimopoulos, Evelina Pappa, Dimitris Niakas, Yannis Tountas

**Affiliations:** 1 Hellenic Open University, Faculty of Social Sciences, Riga Fereou 169 & Tsamadou, Patras 26222, Greece; 2 Center for Health Services Research, Department of Hygiene and Epidemiology, Medical School, University of Athens, 25 Alexandroupoleos St., Athens 11527, Greece

## Abstract

**Background:**

The 12-item Health Survey (SF-12) was developed as a shorter alternative to the SF-36 for use in large-scale studies, particularly when overall physical and mental health are the outcomes of interest instead of the typical eight-scale profile. The main purpose of this study was to assess the validity of the Greek version of the SF-12.

**Methods:**

A stratified representative sample (N = 1005) of the Greek general population was interviewed. The survey included the SF-36, the EQ-5D and questions on socio-demographic and health-related characteristics. SF-12 summary scores were derived using the standard US algorithm. Factor analysis was used to confirm the hypothesized component structure of the SF-12 items. Construct validity was investigated with "known groups" validity testing and via convergent and divergent validity, which in turn were assessed by the correlations with the EQ-5D dimensions. Concurrent validity was assessed by comparisons with SF-36 summary scores.

**Results:**

SF-12 summary scores distinguished well, and in the expected manner, between groups of respondents on the basis of gender, age, education, socio-economic status, self-reported health problems and health services utilization, thus providing evidence of construct validity. Effect size differences between SF-36 and SF-12 summary scores were generally small (<0.2), supporting concurrent (criterion) validity. Significantly lower mean PCS-12 and MCS-12 scores were observed for respondents reporting chronic conditions compared to those without (*P *< 0.001). Convergent and divergent validity were supported by expected relationships with the EQ-5D. Reporting a problem in an EQ dimension was associated with lower SF-12 summary scores, supporting concurrent validity. Sensitivity of the Greek SF-12 and replication of the original measurement and conceptual model were demonstrated.

**Conclusion:**

The results provide evidence on the validity of the Greek SF-12 and, in conjunction to future studies addressing test-retest reliability and responsiveness, support its use in Greek health status studies as a brief, yet valid, alternative to the SF-36.

## Background

In medical research and evaluation, there is an increasing interest in instruments used to measure health-related quality of life (HRQOL) in general population surveys, as well as across a variety of diseases and conditions. HRQOL is a multidimensional concept that includes physical, psychological and social domains of health and is generally accepted as an important outcome measure of health care [1]. The two main approaches to measuring HRQOL are generic and disease-specific instruments, and the majority of experts recommend the use of both concurrently [2]. Regarding the generic instruments, the Short Form Health Survey (SF-36) is probably the one that is most widely used [3].

The SF-36 includes eight dimensions: physical functioning (PF), role physical (RP), bodily pain (BP), general health (GH), vitality (VT), social functioning (SF), role emotional (RE), and mental health (MH) [4]. Each dimension is scored on a 0–100 scale with 0 and 100 corresponding to worst and best HRQOL respectively [5], and the eight dimensions can be summarized in two summary scores of physical and mental health [6], hereinafter referred to as PCS-36 and MCS-36.

The 12-item Health Survey (SF-12) was developed as a shorter alternative to the SF-36 for use in large-scale studies, and its reliability and validity have been documented [7]. Scale scores are estimated for four of the health concepts (PF, RP, RE and MH) using two items each, whereas the remaining four (BP, GH, VT and SF) are represented by a single item. All 12 items are used to calculate the physical and mental component summary scores (PCS-12 and MCS-12) by applying a scoring algorithm empirically derived from the data of a US general population survey [8]. Performance of the component summary scores was initially studied in nine languages and it has been recommended that the US-derived summary scores, which yield a mean of 50 and a SD of 10, be used in order to facilitate cross-cultural comparison of results [9].

The SF-12 has been extensively used in health status studies involving the general population [10-12], as well as in studies with disease groups [13-16]. As for the SF-36, it has been translated into Greek and its reliability and validity were established in a sample of 1007 adults living in the greater Athens area. It was found to have high internal consistency reliability, convergent and discriminative validity and able to distinguish between groups of respondents in the expected manner (known-groups validity) on the basis of gender, age and socio-economic status [17]. Using the same sample, the eight-scale structure of the Greek version of the instrument has been confirmed as well [18].

Recently, the SF-12 (embedded within the SF-36) was administered to a nationally representative sample in a large-scale study aiming to assess the health of the Greek population. The aim of the present study was to examine the psychometric properties of the SF-12 summary scores in terms of the measurement and conceptual model, sensitivity, "known groups" construct validity, convergent and divergent validity and concurrent (criterion) validity and, hence, to increase confidence in using the SF-12 in Greek studies as an alternative to the more time-demanding SF-36.

## Methods

### Sample and data collection

The study was conducted in September 2006 and involved a sample (>18 years old) residing in urban (>2,000 inhabitants) and rural (<2,000 inhabitants) areas of the country and in each of the 13 geographical regions. According to the latest Population Census (2001), the survey population consisted of 8,880,924 individuals. Non-fluent Greek speakers, institutionalized subjects and those incapable of reasoning and decision-making on their own were excluded. Participants were grouped, proportionally to the Greek population, by socio-demographic characteristics according to a three-staged sampling methodology. In the first stage, a random sample of building blocks was selected proportionally to size. In the second, households were randomly selected by systematic sampling. In the third stage an eligible participant was selected by simple random sampling in each household. In total 1,005 willing subjects, out of 1,388 initially approached (response rate 72.4%), agreed to participate and were interviewed face-to-face by trained interviewers. The Research Committee of the Hellenic Open University ethically approved the study and all subjects provided informed consent.

### Survey

Two HRQOL instruments, the SF-36 and the EuroQol EQ-5D, were included in the study. The latter is a two-part, preference-based HRQOL measure, developed by a multidisciplinary transnational consortium of investigators [19]. It addresses five domains: mobility, self-care, usual activities, pain/discomfort and anxiety/depression, with each divided into three severity levels. The second part consists of a vertical 0–100 visual analogue scale (VAS). The EQ-5D has been translated into most major languages, including Greek, and initial evidence on its applicability and adaptability to the Greek environment has been provided [20]. Currently, a large-scale general population study is in progress aiming to demonstrate the construct validity of the Greek version of the instrument. Subjects reported information on socio-demographic variables such as gender, age, marital status, education and employment, with the latter two serving as proxy-estimators of socio-economic status, as information on income could not be reliably collected. Data were also collected regarding various clinical conditions, which are known to be reliable when self reported [21,22]. Utilization of health services such as past-month physician consultations and past-year hospital admissions were also recorded, as they have been shown to be associated with HRQOL [23,24].

### Psychometric properties

The sensitivity of the SF-12 measurement model was evaluated by examining: i) response distributions for each item in order to ensure that the full range of possible responses is used and ii) summary floor and ceiling effects to assess the ability of the items to capture the full range of health states. To ensure that the original conceptual model is satisfactorily replicated, principal components factor analysis with varimax rotation was used, a procedure previously performed in similar studies [13,25]. It was hypothesized that two factors would be obtained. In addition, items originally belonging to the PF, RP, BP and GH domains were hypothesized to load higher on the physical health factor, whereas the VT, SF, RE and MH items were hypothesized to load higher on the mental health factor. However, it has been suggested that VT and SF crossload on both physical and mental components [8] and a crossloading of ≥0.40 is considered to be meaningful [26].

Furthermore, the correlation between physical component items and the PCS score should be higher than with the MCS score and vice versa, i.e. the correlation between mental component items and the MCS score should be higher than with the PCS score [7]. These relationships were examined for the SF-12 items. The proportion of the total variance of PCS-36 and MCS-36 scores explained by PCS-12 and MCS-12 scores respectively was used to assess content validity and the expected standard was ≥90% [8]. This was further evaluated by Pearson's correlation coefficients between SF-12 and SF-36 PCS and MCS scores, and the expected standard was ≥0.9 [8,9].

"Known groups" construct validity was assessed by examining hypothesized relationships between sociodemographic and health-related variables and SF-12 component scores. Specifically, it was expected that females, older subjects, widowed or divorced persons, those with less education and the unemployed would report poorer health [10,11]. It was also expected that those reporting greater use of health services and/or existing clinical conditions would have a lower HRQOL as well [21-24]. Effect size differences between corresponding SF-12 and SF-36 PCS and MCS scores were used to determine if the SF-12 gave similar results to the SF-36 (criterion validity). The effect size difference between SF-36 and SF-12 scores was calculated by dividing their difference by the standard deviation of the SF-36 summary score. To assess the relative magnitude of change, it has been suggested that an effect size of 0.2 is regarded as small, 0.50 as moderate and 0.80 as large [27].

The ability of the SF-12 to discriminate between different levels of health was determined by comparing mean summary scores for subjects reporting no problem, a moderate problem or a serious problem for a given EQ-5D dimension, and it was expected that scores would be higher in the first case [28]. Convergent and divergent validity of the SF-12 were examined via the relationships with the EQ-5D, and it was expected that comparable summary scores and dimensions, e.g. PCS-12 with mobility, self-care, usual activities and pain/discomfort and MCS-12 with anxiety/depression would correlate better, compared to less comparable dimensions. Contrarily, the EQ VAS should correlate reasonably well with both SF-12 summary scores [29].

### Statistical analysis

Data were analyzed using SPSS ver. 13.0 (SPSS Inc., Chicago IL). Summary scores, according to subgroups, were compared with t-Test and ANOVA. Linear regression was performed to determine the total variance of the PCS-36 and MCS-36 scores explained by the SF-12 items. Pearson's correlation coefficient (r) was used to measure the association between SF-12 and SF-36 PCS and MCS scores and between EQ dimensions and VAS with SF-12 summary scores. Correlations >0.50 were regarded as strong [30]. For all tests, statistical significance was assumed for *P *values <0.05.

## Results

The SF-12 item and summary descriptive statistics are presented in Table 1. Four of the items were recoded so that higher scores correspond to better health. Responses were clustered at the upper end of the measurement scale, as could have been expected in a general population. Despite this, the full range of possible responses has been used satisfactorily, supporting the overall sensitivity of the measurement model. The PCS-12 and MCS-12 summary scores were negatively skewed since respondents scored towards the higher end of the health spectrum. However, no floor or ceiling effects were observed, implying that the SF-12 items captured the full range of health states.

**Table 1:** SF-12 item and summary descriptive statistics (N = 1005)

Item description (scale)	Mean (SD)	95% CI	Median	Response frequencies (%)					
				
				1	2	3	4	5	6
Moderate activities (PF)	2.64 (0.66)	2.60-2.68	3.00	10.1	15.7	74.2	-	-	-
Climb several flights (PF)	2.54 (0.72)	2.50–2.59	3.00	13.5	18.5	68.0	-	-	-
Accomplished less (RP)	1.80 (0.40)	1.77–1.82	2.00	20.4	79.6	-	-	-	-
Limited kind of work (RP)	1.82 (0.39)	1.79–1.84	2.00	18.4	81.6	-	-	-	-
Pain interferes^1 ^(BP)	4.36 (1.08)	4.29–4.42	5.00	3.0	7.3	7.2	16.2	66.3	-
Health in general^1 ^(GH)	3.24 (1.10)	3.17–3.30	3.00	5.9	19.9	33.2	26.8	14.2	
Energy^1 ^(VT)	4.19 (1.31)	4.11–4.28	4.00	2.9	9.7	15.6	24.9	30.9	16.0
Social time (SF)	4.16 (1.15)	4.09–4.23	5.00	3.8	7.7	13.3	19.5	55.7	-
Accomplished less (RE)	1.80 (0.40)	1.77–1.82	2.00	20.2	79.8	-	-	-	-
Note careful (RE)	1.84 (0.37)	1.82–1.86	2.00	16.0	84.0	-	-	-	-
Peaceful^1 ^(MH)	3.92 (1.21)	3.84–3.99	4.00	2.3	11.8	20.6	30.4	26.9	8.0
Blue/sad (MH)	4.58 (1.22)	4.50–4.66	5.00	1.6	5.9	9.7	23.5	34.1	25.2
									
Summary statistics	PCS-12	MCS-12							
							
Mean (SD)	49.42 (10.56)	48.91 (9.20)							
95% CI	48.77–50.08	48.34–49.48							
Median	54.21	51.35							
Skewness	-1.34	-1.09							
Minimum (% floor)	14.01 (0.01)	13.89 (0.01)							
Maximum (% ceiling)	66.76 (0.01)	65.39 (0.01)							

^1 ^Item recoded so that higher scores correspond to better health.

Abbreviations: PF: Physical Functioning, RP: Role Physical, BP: Bodily Pain, GH: General Health, VT: Vitality, SF: Social Functioning, RE: Role Emotional, MH: Mental Health

The two-factor conceptual structure of the SF-12 was confirmed (Table 2). Principal components analysis, after varimax rotation, showed that PF, RP, BP and GH items loaded higher on the physical component, whereas RE and MH items loaded higher on the mental component. The VT and SF items expectedly loaded on both components and similar results have been reported elsewhere [7,13]. Correlations (Pearson's r) of individual items and the SF-12 summary scores are also shown in Table 2. Items comprising the PF, RP, BP and GH domains correlated higher with the PCS score, whereas the SF, RE and MH items correlated better the MCS score. These results confirmed the hypothesized item-component correlations, with one exception, namely the VT item appearing to correlate slightly higher with the PCS score.

**Table 2:** Factor structure and item-component correlations of the SF-12

		Factor structure		correlations	
			
Item description	SF-12 domain	Factor 1	Factor 2	PCS-12	MCS-12
Moderate activities	Physical Functioning	**0.84**	0.17	**0.83**	0.31
Climb several flights	Physical Functioning	**0.82**	0.17	**0.81**	0.32
Accomplished less	Role Physical	**0.81**	0.29	**0.83**	0.41
Limited in kind of work	Role Physical	**0.80**	0.28	**0.83**	0.39
Pain interferes	Bodily Pain	**0.72**	0.35	**0.80**	0.45
Health in general	General Health	**0.68**	0.29	**0.72**	0.43
Energy	Vitality	**0.56**	0.39	**0.57**	0.53
Social time	Social Functioning	0.47	**0.56**	0.52	**0.68**
Accomplished less	Role Emotional	0.30	**0.77**	0.37	**0.75**
Note careful	Role Emotional	0.24	**0.76**	0.32	**0.70**
Peaceful	Mental Health	0.11	**0.62**	0.19	**0.64**
Blue/sad	Mental Health	0.27	**0.67**	0.33	**0.75**

Higher loadings of each item on a factor and higher correlations with a SF-12 component are indicated in bold

^1^*P *< 0.01 for all correlations

The PCS-12 and MCS-12 summary scores explained 93.2% and 86.9% of the total variance of the PCS-36 and MCS-36 summary scores respectively (expected standard 90%), supporting content validity of the Greek SF-12. This was further supported by the correlations between SF-36 and SF-12 summary scores exceeding the expected 0.9 standard. Specifically r = 0.97 (*P *< 0.01) between PCS-36 and PCS-12 and r = 0.93 (*P *< 0.01) between MCS-36 and MCS-12. These high correlations are an indication of the validity of the SF-12 scores, with the SF-36 scores acting as criterion variables.

Significant differences were observed within both SF-12 component scores across the distributions of the demographic and health-related variables (Table 3). Men scored higher than women and both summary scores were negatively associated with age. The adopted proxy indicators of socio-economic status (education and employment) were positively related to HRQOL. Furthermore, being divorced/widowed, suffering from a clinical condition and higher health service utilization (physician consultations and hospital admissions) all correlated negatively with PCS-12 and MCS-12 summary scores. These differences were statistically significant (*P *< 0.01) and confirmed expected relationships in support of the construct validity of the instrument.

**Table 3:** Mean (SD) SF-36 and SF-12 summary scores and effect sizes by subgroups

Variable	N (%)	Physical Component Summary (PCS)			Mental Component Summary (MCS)		
			
		SF-36	SF-12	Effect* size	SF-36	SF-12	Effect* size
Total sample	1005 (100)	50.17 (11.80)	49.42 (10.56)	-0.06	47.59 (9.34)	48.91 (9.20)	0.14
Gender							
Male	483 (48.1)	51.71 (11.32)	50.49 (10.27)	-0.11	48.34 (8.66)	49.90 (8.34)	0.19
Female	522 (51.9)	48.74 (12.06)	48.44 (10.75)	-0.02	46.90 (9.88)	47.98 (9.84)	0.11
Age (years)							
18–24	115 (11.4)	56.70 (5.14)	55.25 (4.40)	-0.28	51.00 (5.77)	52.45 (5.71)	0.25
25–34	185 (18.4)	56.47 (5.12)	54.88 (4.38)	-0.31	49.58 (8.07)	51.52 (7.53)	0.24
35–44	180 (17.9)	54.44 (7.27)	53.51 (6.44)	-0.13	48.29 (8.29)	50.18 (8.08)	0.23
45–54	151 (15.0)	51.90 (10.11)	51.10 (9.31)	-0.08	46.49 (10.00)	47.59 (10.00)	0.11
55–64	150 (15.0)	45.87 (13.07)	45.26 (11.68)	-0.05	45.43 (10.77)	47.09 (10.27)	0.15
≥65	224 (22.3)	39.89 (13.28)	40.30 (11.87)	0.03	45.85 (10.25)	46.00 (10.09)	0.01
Education							
<9 years	334 (33.2)	43.28 (13.74)	43.29 (12.21)	0.00	45.12 (10.29)	45.89 (10.27)	0.07
9–12 years	422 (42.0)	53.36 (9.30)	52.13 (8.35)	-0.13	48.34 (8.84)	49.99 (8.55)	0.19
>12 years	249 (24.8)	54.00 (8.21)	53.08 (7.52)	-0.11	49.65 (8.04)	51.12 (7.57)	0.18
Marital status							
Single	244 (24.3)	55.34 (7.99)	54.02 (6.94)	-0.17	49.78 (7.37)	51.35 (7.18)	0.21
Married	646 (64.3)	49.62 (11.77)	48.84 (10.72)	-0.07	47.31 (9.46)	48.71 (9.22)	0.15
Divorced/Widowed	115 (11.4)	42.26 (13.61)	42.97 (11.88)	0.05	44.55 (11.21)	44.85 (11.14)	0.03
Employment							
Working	499 (49.7)	54.41 (7.97)	53.31 (7.05)	-0.14	48.67 (8.06)	50.39 (7.79)	0.21
Retired	227 (22.6)	40.38 (13.98)	40.52 (12.45)	0.01	46.03 (10.72)	46.39 (10.48)	0.03
House keeping	177 (17.6)	48.13 (11.76)	47.65 (10.43)	-0.04	46.36 (10.42)	47.63 (10.59)	0.12
Student	67 (6.6)	55.45 (7.06)	54.10 (6.49)	-0.19	50.38 (7.11)	51.72 (6.33)	0.19
Unemployed	35 (3.5)	53.36 (8.94)	51.72 (8.76)	-0.18	43.21 (10.96)	45.09 (10.30)	0.17
Chronic Disease (1 or more)							
Yes	360 (35.8)	42.45 (13.71)	42.45 (12.29)	0.00	45.07 (10.45)	45.89 (10.46)	0.08
No	645 (64.2)	54.48 (7.78)	53.32 (6.88)	-0.15	49.00 (8.33)	50.59 (7.94)	0.19
Physician Visit (past month)							
Yes	297 (29.6)	43.05 (13.69)	42.95 (12.54)	-0.01	45.55 (10.80)	46.36 (10.61)	0.08
No	702 (69.9)	53.15 (9.43)	52.14 (8.39)	-0.11	48.45 (8.51)	49.97 (8.30)	0.18
Hospitalization (past year)							
None	880 (87.6)	51.29 (10.97)	50.49 (9.71)	-0.07	47.91 (9.03)	49.28 (8.94)	0.15
Once	85 (8.5)	44.29 (14.41)	44.05 (13.12)	-0.02	46.13 (10.38)	46.60 (9.65)	0.05
>Once	30 (3.0)	36.83 (12.89)	36.40 (12.25)	-0.03	40.41 (11.56)	42.13 (11.25)	0.15

* Negative effect sizes indicate a lower SF-12 summary score compared to the respective SF-36 summary score

^1^*P *< 0.01 for all PCS-12 and MCS-12 subgroup differences

SF-12 summary scores were compared to the respective SF-36 components and the concordance between them was noteworthy. Scores were almost identical and, in any case, differences were never greater than two percentage points in any of the subgroups on either the PCS-12 or MCS-12 components, and such a difference would not be subjectively or clinically meaningful [3,5]. Effect size differences between SF-36 and SF-12 scores were generally small (<0.2), implying that the SF-12 gave similar results to the SF-36 and support concurrent (criterion) validity of the Greek version of the instrument.

Significantly lower mean PCS-12 and MCS-12 scores were observed for respondents reporting specific health problems, compared to those without (Table 4). It should be noted that "sleeping disorders" refers to negative responses, from the subjects, to duration- and quality-of-sleep questions, and not to diagnosed insomnia and this perhaps justifies the high prevalence of this condition in the sample. Along with hypertension and obesity, these subgroups contained the largest number of positive respondents, i.e. people reporting the specific health problem and this perhaps implies that the score differences observed in the other disease groups were sometimes insignificant due to the smaller number of people reporting those particular conditions. In any case, the results are indicative of the discriminative ability of the SF-12 since for every health problem, at least one summary score was significantly lower in the group of positive respondents.

**Table 4:** Mean (SD) SF-12 summary scores by self-reported health problems

		SF-12 summary scores	
		
Variable (condition)	N (%)	PCS-12	MCS-12
Diabetes	62 (6.2)	40.84 (12.60)	44.79 (10.98)
Without	943 (93.8)	49.99 (10.17)***	49.18 (9.01)**
Hypertension	134 (13.3)	39.35 (12.30)	45.03 (10.61)
Without	871 (86.7)	50.98 (9.36)***	49.50 (8.81)***
Heart problem	16 (1.6)	34.50 (10.90)	47.81 (11.70)
Without	989 (98.4)	49.67 (10.39)***	48.92 (9.16)
Asthma	15 (1.5)	45.53 (10.72)	48.73 (9.78)
Without	990 (98.5)	49.48 (10.56)*	48.91 (9.19)
Hip/Knee problem	25 (2.5)	43.38 (10.46)	45.39 (9.37)
Without	980 (97.5)	49.58 (10.53)**	48.50 (9.18)
Depression	24 (2.4)	46.52 (9.97)	40.86 (10.58)
Without	981 (97.6)	49.50 (10.57)	49.10 (9.08)***
Sleeping disorders	254 (25.3)	41.10 (12.40)	42.81 (10.66)
Without	751 (74.7)	52.24 (8.13)***	50.97 (7.61)***
Obesity (BMI > 30)	185 (18.4)	44.84 (12.39)	45.95 (9.98)
Without	820 (81.6)	50.46 (9.82)***	49.57 (8.80)***

* *P *< 0.05, ** *P *< 0.01, *** *P *< 0.001

Subjects indicating a moderate or severe problem on any of the EQ-5D dimensions had significantly lower (*P *< 0.001) mean SF-12 component scores compared to subjects reporting no problems, confirming the ability of the SF-12 to discriminate between different levels of health (Table 5). The MCS-12 summary scores appeared to differentiate better than the PCS-12 ones between the three levels in each EQ dimension, except for usual activities where mean scores were quite similar for those reporting moderate and severe problems. On the other hand, the PCS-12 summary scores discriminated better between respondents of the lowest and highest EQ-5D levels (approximately 20 percentage points or more). It should be noted that the number of severe problem reporters in the mobility, self-care and usual activities dimensions was small and could have affected these particular results.

**Table 5:** Mean (SD) SF-12 summary scores by EQ-5D dimensions

		SF-12 summary scores	
		
EQ-5D Dimension	N (%)	PCS-12*	MCS-12*
Mobility			
No problems	790 (78.6)	53.24 (6.60)	50.52 (7.90)
Moderate problems	210 (20.9)	35.44 (10.57)	43.16 (10.90)
Severe problems	5 (0.5)	33.24 (11.17)	34.53 (13.57)
Self-care			
No problems	955 (95.5)	50.38 (9.73)	49.31 (8.84)
Moderate problems	46 (4.6)	31.41 (9.70)	42.03 (11.89)
Severe problems	4 (0.4)	29.43 (7.67)	32.16 (13.95)
Usual activities			
No problems	817 (81.3)	53.08 (6.62)	50.69 (7.76)
Moderate problems	173 (17.2)	33.91 (9.70)	41.28 (10.50)
Severe problems	15 (1.5)	29.06 (9.67)	39.58 (14.19)
Pain/discomfort			
No problems	669 (66.6)	53.87 (6.53)	51.38 (7.64)
Moderate problems	286 (28.5)	41.85 (11.00)	45.18 (9.42)
Severe problems	50 (5.0)	33.34 (11.55)	37.18 (10.79)
Anxiety/depression			
No problems	568 (56.5)	51.78 (8.83)	52.34 (6.88)
Moderate problems	362 (36.0)	47.28 (11.26)	45.73 (8.88)
Severe problems	75 (7.5)	41.95 (13.29)	38.24 (12.05)

* All relationships between EQ-5D dimensions and SF-12 component scores were significant (*P *< 0.001)

Convergent and divergent validity of the SF-12 were confirmed via the relationships with the EQ-5D (Table 6). Comparable summary scores and dimensions correlated better, e.g. PCS-12 with mobility (r = -0.69), usual activities (r = -0.71) and pain discomfort (r = -0.61) and MCS-12 with anxiety/depression (r = -0.47), indicating convergent validity. On the other hand, less comparable summary scores and dimensions correlated weakly, e.g. PCS-12 and anxiety/depression (r = -0.28) and MCS-12 and mobility (r = -0.34), supporting divergent validity of the SF-12. Contrarily, the EQ VAS correlated reasonably well with both SF-12 summary scores, namely r = 0.68 with PCS-12 and r = 0.49 with MCS-12.

**Table 6:** Correlations between SF-12 summary scores and EQ-5D dimensions and VAS

SF-12 summary scores	EQ-5D dimensions					
		
	Mobility	Self-care	Usual activities	Pain/discomfort	Anxiety/depression	EQ VAS
PCS-12	**-0.69**	-0.38	**-0.71**	**-0.61**	-0.28	**0.68**
MCS-12	-0.34	-0.20	-0.40	-0.42	-0.47	0.49

^1^*P *< 0.01 for all correlations

Strong correlations (>0.50) indicated in bold

## Discussion

This study reports on the first ever examination of the psychometric properties of the Greek SF-12 and is expected to add to the growing list of languages and cultures for which the instrument has been evaluated. Initial evidence was provided on the construct and concurrent validity of the instrument, supported by self-reported data on sociodemographic and clinical characteristics. This implies that the SF-12 is potentially suitable for inclusion in large-scale health surveys in Greece and for cross-cultural quality of life comparisons, as a valid alternative to the SF-36.

The embedded form of the SF-12, i.e. as a subset of the SF-36, was used in the present study. It has been demonstrated that both the embedded and stand-alone versions are similar in terms of item ordering, that factor content and structure are equivalent [8] and that responses to the SF-12 items abstracted from the SF-36 are the same as those obtained from the SF-12 administered alone [31]. Perhaps the unembedded form would have been ideal for this study in light of timesaving, however, the use of the embedded form does not pose a threat to the validity of the results.

The two-factor structure of the instrument and the item-factor loadings were confirmed using principal components analysis, thus ensuring that the conceptual model of the original US version was satisfactorily replicated. Hypotheses regarding the correlation of individual item-component correlations were tested and confirmed, except for the VT item which appeared to correlate higher with the PCS-12 than with the MCS-12 score. In general, this was expected since the VT scale is a general measure and usually correlates with both components [6]. Furthermore, VT loaded highly on both summary components. In the cross-cultural context, this particular result has been observed in studies involving general as well as disease populations [7,13,32].

No floor or ceiling effects in the SF-12 scores were observed in this general population sample, indicating the ability of the instrument to capture a full range of health states. Correlations between SF-36 and SF-12 summary scores reached the expected 0.9 standard and the variability in the PCS-36 explained by the PCS-12 and in the MCS-36 explained by the MCS-12 was 93.2% and 86.9% respectively. The concordance between PCS-12 and PCS-36 and between MCS-12 and MCS-36 observed here is in agreement with results from general population studies in the US [7] and Europe [9] as well as with others involving patient populations [13,31,33].

The SF-12 summary scores were able to distinguish between groups of respondents in the expected manner (known-groups validity) on the basis of gender, age, socio-economic status, self-reported health problems and health services utilization (a proxy of HRQOL), providing evidence of its construct validity. The finding that MCS scores decreased with increasing age is not consistent with the majority of the literature that notes that MCS scores tend to improve with increasing age (as opposed to PCS scores which generally decline). A possible explanation for our finding is that 43% and 59% of the 55–64 and >65 age groups respectively reported suffering from multimorbidity, i.e. the co-occurrence of two or more chronic conditions [34], and specifically diabetes, hypertension and heart problems, all of which are clearly associated with impaired HRQOL in all domains [35,36]. In a recent Greek study involving elderly diabetic multimorbid patients, SF-36 subscales hypothetically correlating with the MCS (i.e. VT, SF, RE and MH), were significantly reduced [37]. In another SF-36 study involving a Greek general population, the MCS scores also appeared to decline with increasing age [24].

In a future study specifically aimed at measuring HRQOL, it would be interesting to examine the effect of each sociodemographic and health-related characteristic since, e.g. lower scores for divorced/widowed persons may be due to being older. The same applies for being retired. SF-12 summary scores were compared to SF-36 scores and were found to be very close, within two percentage points at most. These differences are small and unlikely to be of clinical relevance, since it has been suggested that a minimal threshold difference for the SF-36 is around five points [38]. These results, in conjunction with the small effect size differences between the SF-36 and SF-12 scores (<0.2), provide evidence to support the content and concurrent (criterion) validity of the Greek SF-12.

Health conditions, known to be reliable when self-reported, had an effect on SF-12 summary scores and significantly lower mean PCS-12 and MCS-12 scores were expectedly recorded for respondents reporting diabetes, hypertension, heart problems, asthma, hip/knee problems, depression, sleeping disorders or obesity, compared to those without. Using the EQ-5D as a previously tested and accepted standard helped to further support validity. The SF-12 discriminated well between subjects reporting no problem, a moderate problem or a serious problem for a given EQ-5D dimension, since indicating a health problem resulted in significantly (*P *< 0.001) lower mean SF-12 component scores. However, it should be noted that few persons reported severe problems in three of the five domains (i.e. mobility, self-care and usual activities), and this implies that these results should be dealt with cautiously. Finally, convergent and divergent validity of the SF-12 were confirmed by the relationships with the EQ-5D. Comparable summary scores and dimensions correlated higher than less comparable ones, whereas the EQ VAS correlated reasonably well with both SF-12 summary scores.

## Conclusion

Based on the results from this study, the psychometric properties of the Greek SF-12 appear to be sound and suggest its potential for measuring health status in large-scale studies, particularly when overall physical and mental health are the outcomes of interest instead of the typical eight-scale profile. Its major advantage stems from its brevity, which results in fewer burdens for researchers and respondents. It appears to satisfactorily replicate SF-36 summary scores constituting it an attractive generic instrument to use in clinical practice or research when studying HRQOL. In this particular study, cross-sectional construct validity and sensitivity of the Greek SF-12 have been fairly demonstrated. On the other hand, issues such as test-retest reliability, longitudinal construct validity and responsiveness have not been addressed and should be considered for future studies. This is particularly important as health status changes over time and the instrument must be able to detect these changes, particularly those of clinical importance.

## List of abbreviations

BP: Bodily Pain

EQ VAS: EuroQol Visual Analogue Scale

EQ-5D: EuroQol 5-dimension quality of life instrument

GH: General Health

HRQOL: Health-Related Quality of Life

MCS: Mental Component Summary

MCS-12: Mental Component Summary derived from the SF-12

MCS-36 Mental Component Summary derived from the SF-36

MH: Mental Health

PCS: Physical Component Summary

PCS-12: Physical Component Summary derived from the SF-12

PCS-36: Physical Component Summary derived from the SF-36

PF: Physical Functioning

RE: Role Emotional

RP: Role Physical

SF: Social Functioning

SF-12: Short Form 12-item Health Survey

SF-36: Short Form 36-item Health Survey

VT: Vitality

## Competing interests

The author(s) declare that they have no competing interests.

## Authors' contributions

**NK **was responsible for analyzing and interpreting the data and drafting the manuscript. **EP **assisted in the statistical analysis. **DN **designed the study and revised the manuscript for intellectual content. **YT **was responsible for conception of the study. All authors have read and approved the final manuscript.
